# CAN DO Houston: A Community-Based Approach to Preventing Childhood Obesity

**Published:** 2010-06-15

**Authors:** Nancy Post Correa, Beverly Jean Gor, Nancy G. Murray, Christine A. Mei, William B. Baun, Lovell Allan Jones, Nicole B. Hare, Deborah Banerjee, Toral F. Sindha

**Affiliations:** University of Texas Health Science Center at Houston, Houston, Texas; University of Texas M. D. Anderson Cancer Center; University of Texas Health Science Center at Houston, Houston, Texas; Coca-Cola North America (Minute Maid Business Unit), Houston, Texas; University of Texas M. D. Anderson Cancer Center, Houston, Texas; University of Texas M. D. Anderson Cancer Center, Houston, Texas; City of Houston, Houston, Texas; Deborah Banerjee; Houston Department of Health and Human Services, Houston, Texas; Houston Department of Health and Human Services, Houston, Texas

## Abstract

**Background:**

Comprehensive, community-based efforts may reduce rates of childhood obesity.

**Community Context:**

Almost half of the children in Houston are overweight or obese, even though Houston has many available resources that support good nutrition, physical activity, and prevention of weight gain among children.

**Methods:**

We used existing resources to implement a community-based, childhood obesity prevention initiative in 2 low-income neighborhoods in Houston. On the basis of input from community members, we coordinated various activities to promote healthy living, including after-school programs, grocery store tours, wellness seminars, cooking classes, and staff wellness clubs.

**Outcome:**

Preliminary findings indicated that residents in the communities are using additional opportunities to participate in physical activity and nutrition education.

**Interpretation:**

Implementing a successful childhood obesity prevention initiative in an urban setting is feasible with minimal funding through the use of existing resources.

## Background

The negative consequences of childhood obesity have been well documented (1), yet almost one-third of US children remain overweight or obese (2-4), and Houston, Texas (Harris County), is no exception to this trend. In 2007, 27% of fourth-grade children were classified as obese, having a body mass index (BMI) at or above the 95th percentile, and an additional 19% were classified as overweight (BMI ≥85th to <95th percentile) (5).

Some community-based initiatives have helped children lose weight (6) and prevented them from gaining weight (7), but most have failed to demonstrate long-term community-wide reductions in childhood obesity rates (8,9). Obesity prevention experts recommend a comprehensive approach, involving schools, parks, health departments, community programs, families, and health care practitioners (1,10,11). We conducted a pilot project that relied on the principles of community engagement and translational research to help nonprofit organizations, health practitioners, and researchers coordinate efforts to translate evidence-based practices to prevent childhood obesity in the community (12-14). Although building strong community partnerships can be time consuming, doing so can increase community trust, community ownership, and the relevance of the intervention to ensure it fits in the context of the community (15,16).

Our initiative, CAN DO Houston (**C**hildren **A**nd **N**eighbors **D**efeat **O**besity; la **C**omunidad **A**yudando a los **N**iños a **D**errotar la **O**besidad) has a mission to prevent and decrease the rates of childhood obesity in the Houston metropolitan area through physical activity, nutrition, and healthy minds by enabling the broadest collaboration of people, institutions, organizations, and local government. CAN DO Houston encourages communities to identify and prioritize initiatives to promote physical activity, good nutrition, and healthy minds and to support identified initiatives to help reduce rates of childhood obesity. The CAN DO Houston pilot initiative began in August 2008 and was conducted in 2 Houston neighborhoods.

## Community Context

Houston, Texas, is the fourth-largest city in the United States (17); it has more than 2 million residents and covers 634 square miles (18,19). Houston's racial/ethnic profile (49% white, 37% Hispanic, 25% African American, and 5% Asian) is diverse (20). In 2007 in Harris County, 27% of residents aged 18 years or older were classified as obese (BMI ≥30 kg/m^2^), and in the Houston metropolitan statistical area (10 counties that surround Houston), 9% reported they had been diagnosed with diabetes (21). In 2008, 7% of residents aged 18 years or older reported they had been diagnosed with cardiovascular disease, and 29% reported they had been diagnosed with high blood pressure (22).

Houston is divided into 88 geographically designated areas, referred to as *super neighborhoods*, in which residents are encouraged to work together to identify, plan, and set priorities to address the needs and concerns of the community (18). Sunnyside and Magnolia Park are 2 of Houston's super neighborhoods and the locations of the CAN DO Houston pilot initiative. Sunnyside is the oldest African American community in Houston and has 18,629 residents. According to the 2000 census, 94% of Sunnyside residents are African American, 38% earn less than the poverty level, and less than half are employed (23). Magnolia Park has 21,302 residents, of whom 96% are Hispanic, 31% earn less than the poverty level, and less than half are employed (24).

After identifying the Sunnyside and Magnolia Park neighborhoods as potential sites for the CAN DO Houston pilot, CAN DO Houston stakeholders identified more than 60 programs in Houston that addressed childhood obesity. On the basis of this large number of programs, CAN DO Houston hypothesized that it could support the prevention of childhood obesity with minimal funding by engaging with communities, coordinating with existing organizations, and using available resources.

## Methods

### Formation of CAN DO Houston

After *Men's Fitness* magazine named Houston the "Fattest City in America" in 2005, the office of the mayor initiated the Mayor's Wellness Council (MWC) to encourage and motivate Houstonians to make wise choices regarding healthy eating and regular physical activity through education and participation in fun activities (25). To sustain this vision, in 2006 the MWC created the Houston Wellness Association (HWA), a nonprofit association to engage businesses and the wellness industry in efforts to increase the wellness of all Houston citizens (26).

In October 2007, the HWA and the MWC invited interested stakeholders to address the problem of childhood obesity in Houston. Through the informal networks of HWA and MWC members, momentum and interest began to grow, and a large consortium of stakeholders — including city services, experts in health disparities and childhood obesity, pediatricians, universities, and community programs — coordinated efforts to create a comprehensive, community-based childhood obesity prevention program. Furthermore, in 2006 the National Institutes of Health funded the Center for Clinical and Translational Sciences (CCTS) at the University of Texas Health Science Center at Houston as one of the first 12 Clinical and Translational Science Awards, which gave staff resources to coordinate the CAN DO Houston collaborative.

Interested stakeholders initially met monthly, and as plans became more concrete, several committees (executive, community engagement, programming, evaluation, data gathering, communications, and development) were formed. The executive committee became the board of directors when CAN DO Houston became an independent 501(c)(3) nonprofit organization. CAN DO Houston committees continued to meet monthly, the entire consortium met quarterly, and monthly e-mail updates were sent to keep all partners informed and engaged. A timeline of the formation of CAN DO Houston is presented in Table 1. Because this was a community action project, institutional review board approval was not required.

### Identification of super neighborhoods

In partnership with the City of Houston Department of Health and Human Services (HDHHS), CAN DO Houston identified the super neighborhoods of Sunnyside and Magnolia Park as the pilot sites for CAN DO Houston. (HDHHS had assessed health and wellness in these 2 neighborhoods in 2007 by interviewing a representative sample of residents in each neighborhood through a stratified cluster sampling method, and it shared the data collected to assist in the development of CAN DO Houston.) Once the 2 neighborhoods were selected, an elementary school and a park in each neighborhood were identified to be the anchors of CAN DO Houston. Elementary school students, aged 6 to 12, were identified as the primary focus of the pilot. The secondary focus was on parents, families, and school staff because their actions can indirectly affect children's weight. For example, parents' food purchases affect the types of foods children have access to, and school staff can influence how much time students spend being physically active.

### Key informant interviews and focus groups

Eight key informant interviews were held with the school principals, park managers, physical education teachers, staff of the Metropolitan Transit Authority of Harris County (METRO), and police officers to prioritize the needs for each community. Interviewees were asked to describe strengths and barriers in their community in accessing physical activity and good nutrition and in developing healthy minds. They were also asked to identify and prioritize initiatives that promote physical activity, good nutrition, and healthy minds. Interview participants were identified as either being in key roles to support CAN DO Houston's mission (principals and physical education teachers) or people recommended by their organization (METRO staff and police officers). Both the METRO staff and the police officers expressed a willingness to support CAN DO Houston through promoting initiatives and increasing police presence around the schools and parks. We also conducted focus groups with parents in each neighborhood. In Magnolia Park we received permission from the principal to attend an existing parent meeting. In Sunnyside, we incorporated a focus group into 1 of our parent education sessions. CAN DO Houston was also introduced at a community meeting in Sunnyside and Magnolia Park through the Houston Police Department. The interviews lasted approximately 60 minutes and the focus groups 30 minutes.

### Literature review

We reviewed the literature for evidence-based practices and target behaviors that prevent weight gain in children, including the *Expert Committee Recommendations Regarding the Prevention, Assessment, and Treatment of Child and Adolescent Overweight and Obesity* (1). We selected 5 of the evidence-based target behaviors and created key messages to serve as the focal point of the CAN DO Houston initiatives: eat 9 fruits and vegetables daily, eat breakfast daily, limit screen time to no more than 2 hours daily, engage in moderate to vigorous physical activity for 60 minutes daily, and spend 60 minutes of uninterrupted family time together daily. (The fifth key message was adopted from the target behavior that families should eat together at mealtime and modified to fit the context of our communities in which many parents are not able to be home during mealtimes. It also supports our focus of developing healthy minds.)

### Assessment of partner capacity

After reviewing the HDHHS assessments and after completing key informant interviews, focus groups, community meetings, and review of the literature for evidence-based practices, we also assessed the capacity of our partners. With CCTS support, CAN DO Houston stakeholders created a public resource database of the 60 local programs that address childhood obesity and made it accessible online (http://ccts.uth.tmc.edu/ccts-services/resource-lists). As we prioritized initiatives, it was necessary to implement initiatives that the community requested (to develop community trust), that were evidence-based and sustainable (to increase the likelihood of success), and that were not burdensome to the limited capacity of the CAN DO Houston partnership. (For example, the Magnolia Park elementary school requested a second physical education teacher, so its students could spend more time in physical education each week. Although we supported students spending more time in physical education, we concluded that funding a teacher's salary was beyond the capacity of our organization.)

### Findings of key informant interviews and focus groups

In Magnolia Park, participants of the key informant interviews and focus groups indicated that children had good access to healthy eating resources. Fresh produce was available and reasonably priced at local grocery stores. The school had a community garden, and students received cooking classes and nutrition lessons through a partnership with a local nonprofit organization, Recipe for Success. The school was also trained in CATCH (Coordinated Approach to Child Health), an evidence-based coordinated school health program (27).

Participants identified lack of physical activity as the primary barrier to preventing childhood obesity. Some parents were reluctant to categorize their children as overweight and obese and preferred to promote child health instead of addressing weight management. Several mothers expressed a concern for their child's safety off campus. The elementary school principal said that most children went home after school and had minimal opportunities for physical activity because of lack of awareness of existing opportunities, transportation barriers, and fear of crime. The principal expressed a need for a second physical education teacher, a general after-school program, an after-school soccer program, and staff wellness programs. The physical education teacher also expressed a need for another physical education teacher and more equipment. Parents expressed interest in aerobics classes.

Parents expressed concerns about park safety, and the local park manager expressed a need for transportation to transport the students to the park after school. The city park was 0.4 mile from the elementary school and offered a free after-school program that provided students with an opportunity to engage in safe, supervised physical activity. However, a busy 4-lane street and a bayou prevented most parents from allowing their children to walk to the park.

In the Sunnyside neighborhood, results from the assessments, focus groups, key informant interviews, and community meetings indicated that children were receiving more than the recommended 60 minutes of moderate-to-vigorous activity each day. Students participated in physical education 4 times per week during the school day. Many of the students walked to school and attended the after-school program conducted by the City of Houston Parks and Recreation Department, and the school was also trained in CATCH. The park was directly across the street from the school, making it easy for the students to access the park after school.

Participants of the key informant interviews and the focus groups identified nutrition education as the primary need to prevent childhood obesity in the Sunnyside community. Specifically, the principal requested parent education, grocery store tours, and a community garden; the park manager requested resources to teach nutrition education; and the physical education teacher requested parent education and equipment to measure the heights and weights of students. Parents identified neighborhood safety as their biggest concern.

## Outcome

### Outcomes of key informant interviews and focus groups

On the basis of participants' concerns about park access for children in the Magnolia Park neighborhood, we partnered with the local park recreation staff and arranged for them to conduct an after-school program at the school twice per week from 2:45 pm to 5:00 pm. The park staff led the activities, and CAN DO Houston provided volunteers who worked more than 160 hours to assist the park staff and supervise the students (Table 2). More than 80 students signed up for the program, which demonstrated that transportation to the park may be a substantial barrier for students participating in the park's after-school program. Because of the success of the pilot, the school district agreed to provide bus transportation between the school and the park during the 2009-2010 school year.

More initiatives were created for Magnolia Park (Table 2) and primarily promoted through announcements that were sent home to parents by the school. The school office staff was also well informed of the CAN DO Houston activities and answered questions from the students and parents. To receive feedback on the CAN DO Houston initiatives, CAN DO Houston partners formally met with key stakeholders in Magnolia Park 3 times per year. In addition, CAN DO Houston partners spent ample time in the communities, and feedback was generated through informal conversations with parents, students, and staff.

On the basis of participants' request for more nutrition education for children in the Sunnyside neighborhood, we coordinated a monthly wellness seminar to educate parents on good nutrition and various wellness topics. Grocery store tours were also offered and focused on how to buy healthy foods on a budget. A nutrition carnival was hosted at the park's after-school program to educate students about healthy eating, and we provided the park with supplies to incorporate nutrition education into its after-school program, in addition to other efforts (Table 3).

The Sunnyside initiatives were promoted through a weekly letter from the principal that went home to parents and through announcements to the parents of the students in the park's after-school program. Feedback was also received in the Sunnyside community through formal meetings 2 times per year and informal conversations with staff, students, and parents.

CAN DO Houston community partners organized their own wellness initiatives during the pilot, in which CAN DO Houston provided support. In Magnolia Park, teachers started a running and walking club for students and staff before school and coordinated bus transportation to transport students to a celebratory 1-mile run with a local program, Marathon Kids. In the Sunnyside neighborhood, teachers had students read a daily food fact during morning announcements. A community garden was also planted at the Sunnyside park.

### Other outcomes and feedback

Results from the CAN DO Houston initiatives were encouraging. More than 400 students and family members attended the "fitness explosion" (an effort to increase awareness about opportunities to engage in physical activity), an average of 48 students attended each after-school program, the after-school soccer program was filled to capacity at 40 students, and more than 120 students attended the nutrition carnival. Furthermore, more than 100 people volunteered at CAN DO Houston events for a total of 450 recorded volunteer hours. Feedback from students who participated in CAN DO Houston was obtained by volunteers who asked small groups of children questions during one of the last sessions of the after-school program. Their comments demonstrated the positive effects the pilot had on students' attitudes and behaviors (Table 4).

The school district provided access to student physical fitness assessments and BMI measurements, which will allow us to compare the BMI of students in the CAN DO Houston schools to that of students in similar schools that do not participate in CAN DO Houston.

The pilot initiative successfully formed a consortium of people and organizations interested in addressing childhood obesity. More than 70 organizations and 100 people participated in the development of CAN DO Houston, including the office of the mayor, METRO, the Houston Police Department, academic institutions, law firms, public relations firms, members of the media, foundations, corporations, government, city services, physicians, nonprofit organizations, the school system, and the city parks and recreation department. Because of the quarterly CAN DO Houston meetings, the monthly CAN DO Houston committee meetings, and the online database, organizations are more aware of other organizations in the community, and we have informally observed more organizations working together. These collaborations could have a substantial effect on the community, as most of our partner organizations are conducting work that is not limited to the super neighborhoods of Sunnyside and Magnolia Park.

### Program challenges

CAN DO Houston overcame various challenges during its development and implementation. The largest barrier was reaching parents, despite offering activities that the parents requested. Language barriers and work schedules may have prevented parents from being more active in the initiative. In retrospect, we may have increased parent participation by offering activities at different times of the day, advertising more, and offering incentives for parent participation.

Furthermore, identifying which initiatives to support has been challenging. Community requests do not always align with the recommendations from the expert committee (1). To increase the likelihood of success, implementing evidence-based practices must be balanced with developing trust and a true partnership with the community (16). To address this challenge, we created a program governance committee to create guidelines on how to prioritize the initiatives we support.

Finally, although the pilot successfully used existing resources to offer opportunities for increased physical activity and nutrition education, we were limited by lack of funding in what we could provide the communities. All staff time and materials — with the exception of the kick-off events and the equipment, which was provided by a MWC grant from the Texas Governor's Advisory Council on Physical Fitness — were donated in-kind by our partners. CAN DO Houston will continue to support the super neighborhoods and their residents during the gradual move toward ownership of their health. However, additional funding would allow CAN DO Houston to offer more activities, expand to additional neighborhoods, and complete a comprehensive evaluation. We would also like to support more initiatives based on the third goal of CAN DO Houston — developing healthy minds — through supporting good decision-making skills and positive family and community relationships.

## Interpretation

To determine the effectiveness of CAN DO Houston, a comprehensive evaluation is necessary. However, our preliminary data indicate that children in the 2 communities are taking advantage of additional opportunities to participate in physical activity and receive nutrition education. These results suggest that it is possible to use existing resources to improve children's health.

The pilot initiative demonstrated that by engaging communities, collaborating with organizations, and using existing resources, it is possible to provide communities with better access to a healthy lifestyle with minimal funding. However, the ability to succeed without funding may depend on available resources in a large city, making it difficult to replicate in smaller communities. For example, without the support of substantial staff time from the CCTS and others, such as the Center for Research on Minority Health at the University of Texas M. D. Anderson Cancer Center, coordinating the CAN DO Houston initiatives may not have been possible. Furthermore, CAN DO Houston was implemented in 2 neighborhoods that the HDHHS had previously partnered with to conduct health assessments. Because building community partnerships and trust takes time, the previous partnership between the super neighborhoods and HDHHS may have made the communities more willing to partner and to participate in initiatives that promote good health (15,16).

Many factors contributed to the success of the pilot of CAN DO Houston. Some of the factors that we believe were critical to our success were

- obtaining the mayor's support of CAN DO Houston (which was helpful when we approached new organizations about partnering);
- identifying a common vision and goals among partners;
- building an infrastructure that provided many opportunities for partner ownership;
- communicating regularly with partners and the communities, including spending ample time in the communities;
- partnering with both senior-level people and "on the ground" people in an organization (which helped us create the strongest partnerships);
- remaining positive and enthusiastic when interacting and problem solving with partners, volunteers, and the communities;
- and engaging and listening to the communities and allowing the communities to prioritize the initiatives. (Collaborating with communities allows assets and resources in the community to be built on and strengthened [28].)

We believe that the CAN DO Houston pilot initiative demonstrated progress in improving the health of the community by disseminating evidence-based practices through community engagement. The CAN DO Houston board of directors is seeking funding to expand to additional neighborhoods, strengthen continued support to our current neighborhoods, and facilitate a more comprehensive evaluation of the effectiveness of our initiative.

## Figures and Tables

**Table 1:** Timeline of the Development of CAN DO Houston, Houston, Texas

Date	Event or Activity
September 2007	The Community Advisory Board of the CCTS identifies childhood obesity as the primary health concern in the Houston community.
October 2007	The Children and Family Wellness Committee of the HWA convenes for the first time and decides to implement a community project that focuses on helping prevent childhood obesity.
	The Childhood Obesity Taskforce of the MWC convenes for the first time and announces it will draft a white paper regarding childhood obesity in Houston.
February 2008	The MWC, the HWA, and the Community Advisory Board of the CCTS decide to work collaboratively to address childhood obesity in the Houston community as CAN DO Houston.
April 2008	The first formal CAN DO Houston meeting is held with all interested stakeholders. Representatives from key organizations such as the school district, parks and recreation, METRO, Houston Police Department, and the City of Houston Department of Health and Human Services are invited to attend. Seven committees are created: executive, community engagement, programming, evaluation, data gathering, communications, and development.
May 2008	Contact is initiated with key community stakeholders, such as park managers, school principals, physical education teachers, and parent groups to assess and prioritize initiatives for the pilot.
May 2008-August 2008	CAN DO Houston stakeholders meet each month with all 7 committees. Plans move forward to implement CAN DO Houston in fall 2008.
September 2008	The pilot of CAN DO Houston is implemented in 2 Houston super neighborhoods.
	CAN DO Houston becomes an independent 501(c)(3) nonprofit organization.

Abbreviations: CAN DO, Children and Neighbors Defeat Obesity; CCTS, Center for Clinical and Translational Sciences; HWA, Houston Wellness Association; MWC, Mayor's Wellness Council; METRO, Metropolitan Transit Authority of Harris County.

**Table 2:** CAN DO Houston Initiatives in the Magnolia Park Neighborhood, Houston, Texas

Initiative	Outcome
In partnership with City of Houston Parks and Recreation, we offered an after-school program at the Magnolia Park elementary school. Park staff led activities such as soccer, kickball, sack races, relay races, and other activities that encouraged physical activity.	The program was offered twice a week for 7 weeks for a total of 14 sessions. Attendance ranged from 31 students to 63 students per session with an average number of 48 students and 8 volunteers at each session. Each session was 2.25 hours long.
After the completion of the after-school pilot, the elementary school and the park continued to partner and offer soccer after school. During the sessions the students participated in soccer drills and a game.	The program was offered once per week during the spring semester, limited to 40 students, and was filled to capacity. Each session was 2 hours.
In partnership with Minute Maid, we provided pretzels and 100% juice for the students in the after-school program.	All students in the after-school program were offered pretzels and 100% juice. Typically, all participants ate the snack.
In partnership with M. D. Anderson Cancer Center Wellness Programs, we initiated a staff wellness committee at the Magnolia Park elementary school. School staff addressed wellness initiatives at the school and participated in stress management activities.	Seven teachers and faculty met biweekly. Activities included planning a school field day and participating in visualization techniques, yoga exercises, breathing exercises, walking exercises, and stress management activities.
In partnership with Recipe for Success, we offered after-school cooking classes for pairs of students and parents. During each session, students gathered fresh vegetables and herbs, such as radishes and basil, from the school garden. Some of the meals prepared by the students and parents included homemade wheat pasta with vegetables and pesto, roasted vegetable sandwiches, tabouli, and whole wheat pizza.	A total of 4 sessions were offered, and each session lasted 2 hours. A total of 42 students and parents attended the after-school cooking classes (an average of 10 people attended each session).
In partnership with the MWC, we provided the school with equipment, including 20 soccer balls, soccer goals, and hula hoops, to increase opportunities to engage in physical activity.	The soccer equipment was used twice per week during the after-school program. The physical education teacher reported that the equipment was also used during physical education classes and that students requested the equipment during recess.
In partnership with the University of Texas School of Public Health, we provided the teachers with booklets and training on how to incorporate physical activity in the classroom. The training lasted for 30 minutes, and the booklets included 15 activities that could be done in a limited space, such as the classroom, to get students physically active in short periods of time (5-10 min).	Twenty teachers attended the training, and 35 teachers received booklets. The teachers indicated that they used the activities and that the students enjoyed them.
In partnership with the MWC, we coordinated the efforts of many local nonprofit organizations to offer a "fitness explosion" to increase awareness of opportunities to engage in physical activity. Some of the local organizations that attended included the local fire station, the Houston library, the City of Houston Department of Health and Human Services, and the MWC.	Approximately 400 students, family members, and teachers attended the 2-hour event. Students participated in various physical activity stations and visited booths with information on local resources that support healthy living. The students had to participate in 5 physical activity stations, such as shooting a ball in a goal and jumping rope, to receive a prize (a CAN DO Houston cinch bag).
In partnership with the University of Houston, college students organized a field day for the students.	Approximately 175 students and staff participated in a 2.5-hour field day. Students and staff engaged in various activities that required physical activity, such as obstacle courses, tug of war, relay races, and basketball shooting.

Abbreviations: CAN DO, Children and Neighbors Defeat Obesity; MWC, Mayor's Wellness Council.

**Table 3:** CAN DO Houston Initiatives in the Sunnyside Neighborhood, Houston, Texas

Initiative	Outcome
We coordinated speakers for "Coffee on the Run," a monthly presentation on wellness. Parents attended the 30-minute morning sessions after dropping their children off at school. Topics included nutrition 101, the link between physical activity and academic performance, and relaxation techniques.	The wellness talks were offered monthly, and on average 15 parents attended each session.
In partnership with Baylor College of Medicine, we provided a scale and a stadiometer to evaluate the height and weight of all participants of the CAN DO Houston pilot.	The elementary school was able to record heights and weights for the 404 students at the school. This allowed them to meet the state mandates on reporting height and weight. The school also reported that the teachers were using the scales to monitor their own weight.
In partnership with the University of Texas School of Public Health, we provided teachers with booklets on how to incorporate physical activity in the classroom. The booklets included 15 activities that could be done in limited space, such as the classroom, to get students physically active in short periods of time (5-10 min).	Thirty-five teachers received booklets.
In partnership with local grocery stores and the City of Houston Department of Health and Human Services, we offered grocery store tours. Dietitians taught parents how to buy healthy foods in their local grocery stores on a budget. Participants were able to take home fresh produce. Activities focused on reading nutrition labels; choosing frozen, canned, or fresh produce; identifying the differences between brand-name versus store-brand products and low-fat versus whole-fat dairy products; and knowing the minimum daily fiber intake.	Two grocery store tours were offered in the Sunnyside community. Seventeen Sunnyside residents attended the first tour, and 13 attended the second tour. Each tour lasted 90 minutes.
We coordinated the efforts of many local nonprofits to offer a "nutrition carnival" to provide various interactive activities for the students to learn about healthy eating. Topics included descriptions of the Food Guide Pyramid, portion size, and sugar content in beverages. Participating organizations included the City of Houston Parks and Recreation, the University of Texas School of Public Health, the Children's Museum, and the Houston Area Dietetic Association.	Approximately 120 students attended the 2-hour event.
In partnership with the University of Houston, college students organized a field day for the students.	Approximately 120 students and staff participated in a 2.5-hour field day. Students and staff engaged in various activities that required physical activity, such as a hula-hoop challenge, a basketball obstacle course, and a running relay.
In partnership with the Mayor's Wellness Council, we provided the park with additional equipment, such as jump ropes and cones, to support physical activity	The park reported using the equipment regularly.

Abbreviation: CAN DO, Children and Neighbors Defeat Obesity.

**Table 4:** Student Feedback From CAN DO Houston, Houston, Texas

**Question**	Student Response
What do you like about CAN DO Houston?	"It lets you express yourself, like running everywhere . . . but it still has rules . . . you can just be happy, play games, get active, and stay fit. . . . You know . . . being healthy."
	"Being active . . . helps children slim down."
	"It helps you exercise."
	"It has gotten me into a habit of working out."
	"I like everything about CAN DO."
	"I like when we do activities, like soccer."
What would you be doing if you weren't in CAN DO Houston?	"Being bored and watching [television]."
	"Watching [television]."
How do you feel after CAN DO Houston?	"Happy, I never get this much time to play outside at home."

Abbreviation: CAN DO, Children and Neighbors Defeat Obesity.
